# Medical and Philosophical Intelligence

**Published:** 1815-08

**Authors:** 


### MEDICAL AND PHILOSOPHICAL INTELLIGENCE.

*ROYAL SOCIETY.*—On Thursday, the 25th May, a paper by Dr. PARRY was read, on the cause of the pulsation of the arteries. He stated the opinion of Haller, which is generally received by physiologists, and that of Bichat, who had rejected Haller's explanation in consequence of his dissections of living animals. Dr. Parry then stated the results which he himself had obtained by laying open the arteries of living sheep and rabbits. No alteration in the size of the artery could be perceived, but a motion of the artery backwards and forwards, corresponding to the inspiration and expiration of the animal. Dr. Parry conceives that the artery is a tense tube always full of blood, and that, when its diameter is diminished by external pressure, the blood makes an effort to restore the original size. Hence the pulsation.—That the artery is always always full of blood cannot be questioned, but it is not less certain that it possesses a contractility, by which its bulk is accommodated to its contents. We wish Dr. Parry, instead of consulting the visionary theories of the continent, had paid some attention to the acknowledged correctness of our own island. Where could he meet with experiments more correct, and inferences so conclusive, as in Mr. Hunter's treatise on the Vascular System?

At the same meeting, part of a paper by Mr. Donovan was read, giving an account of a new vegetable acid, discovered by him in the juice of the berries of the sorbus aucuparia, together with some observations on malic acid. He extracted the juice of the ripe berries by pressure, precipitated by acetate of lead, washed the precipitate in boiling water, and threw the whole upon the filter. A hard white mass remained upon the filter, and the liquid which passed through deposited, on cooling, fine, white, silky, needle-form crystals. Scheele had stated the acid in these berries to be the malic. But malate of lead had never been observed to crystallize. To clear up the subject, Mr. Donovan saturated the juice of ripe apples with potash, and precipitated the liquid by acetate of lead. The precipitate treated with boiling water, as in the preceding case, yielded similar silky crystals; but the substance remaining on the filter was a soft magma. Gooseberry juice treated in the same way yielded no crystals; nor raspberry juice, nor the juice of elder berries, nor of sedum tectorum, nor green apples. Thus it appears, that acetate of lead precipitates two different acids in the first two liquids. The first an acid not hitherto observed, which readily forms a supersalt with oxide of lead, soluble in hot water; and this solution on cooling deposits the neutral salt in silky crystals: the second malic acid, which forms with oxide of lead a salt not capable of crystallizing. To obtain the new acid in a state of purity, the colourless silky crystals are to be treated with a quantity of sulphuric acid capable of saturating the greater part, but not the whole, of the oxide of lead present. The liquid being filtered to separate the sulphate of lead, a current of sulphureted hydrogen is driven through it till the whole remaining oxide of lead is thrown down. The filtered liquid is now boiled for some time, and then exposed to the air for a few days to get rid of the sulphureted hydrogen.

On Thursday, the first of June, Mr. Donovan's paper was continued. To the acid thus obtained he gave the name of *sorbic* acid. It possesses the following properties. It is colourless. Its taste is intensely sour, and it reddens vegetable blues. It does not crystallize. It does not readily undergo spontaneous decomposition. Mr. Donovan kept a quantity of it in a phial for a year; no other change happened except the deposition of a very small quantity of mucilaginous matter. It combines with oxide of lead in three proportions, forming, 1. Subsorbate of lead, which is a hard white insoluble powder. 2. Sorbate, which may be obtained either in powder or in crystals, and which is likewise insoluble. 3. Supersorbate, which does not crystallize. The alkaline supersorbates may may be all obtained in the state of crystals. It forms soluble salts with barytes, lime, and magnesia. It does not combine with alumina. These properties sufficiently distinguish this acid from the malic.

Mr. Donovan likewise related his experiments on the preparation of malic acid. He found none of the methods recommended by Scheele capable of furnishing pure malic acid. He considers Vauquelin's process for preparing malic acid from the juice of the sedum tectorum as the only one that yields a tolerably pure acid.—We may observe here, that about ten years ago some experiments were made on the preparations of malic acid, and it was found that, unless it be freed from mucilage before precipitation with lead, it cannot afterwards be separated from a considerable quantity of gummy matter which seems to fall down in combination with the lead. Most of the difficulties which have occurred to chemists in preparing this acid are ascribed to not animadverting to this circumstance.

Mr. Donovan conceives it likely that the bitter principle which exists at first in various fruits and disappears as they advance to maturity, may be the basis of some of the vegetable acids.

At the same meeting, a paper by Sir Everard Home, bart. on the respiratory organs of some genera of vermes that live in water, was read. These organs consist of a number of openings on both sides of the neck, which lead into spherical or flattened balls. Water passes through these openings into the bags, and is afterwards thrown out again. The reason why the water does not enter at the mouth as in fishes, is because these animals, as the leech, require their mouths for suction, either to procure food or to fasten themselves to other bodies.

On Thursday, the 15th of June, a paper by Sir Everard Home, bart. was read, on the mode of generation of the lamprée and myxine. He found by a great many dissections at differents periods during the summer, that these animals are all hermaphrodites; those which were supposed to be males, producing eggs as well as the supposed females.

At the same meeting, a paper by Anthony Carlisle, Esq. was read, on the connection between the extravascular and vascular parts of animals. Hair, feathers, nails, hoofs, are extravascular substances, and possess no vessels. The chief object of the paper was to shew, that the shells of shell-fish and snails are likewise without vessels. They cannot be injected. Their membranes do not exhibit the same appearance as those that contain vessels. When a piece of snail-shell was broken off, the injury was repaired by a viscid substance applied internally, and then layers of calcareous matter were laid over it.

At the same meeting, a paper by John George Children, Esq. was read, on the effects of a very large galvanic battery. It consisted of twenty pair of zinc and copper plates, six feet long and two feet six inches broad, joined together by straps of lead and plunged in a mixture of nitric and sulphuric acids, diluted with from twenty to forty times their weight of water. By this battery metallic tallic wires were ignited in the following order, beginning with the wire most easily ignited.

| | |
|---|---|
| Platinum | Copper |
| Iron | Silver |
| Gold | Zinc. |

Tin and lead are so fusible, that with them the experiment could not be tried. Mr. Children considers the ignitability as the inverse of the conducting power of the metals; therefore platinum conducts worst and the zinc best of the above six metals. When the two poles of the battery were connected by two parallel platinum wires of different sizes, the thick wire was ignited and not the fine one; but, when the two wires were tied one to the end of the other, the fine wire was ignited first.

Iron wire was slit, some diamond powder put into the slit, and this powder surrounded by iron wire above and below. The wire was faintly ignited. The diamond powder disappeared and the iron was converted into steel and partly fused. This demonstrates the truth of Clouet's original experiment, which was afterwards verified by Sir George Mackenzie. Iridium was fused by the battery and reduced to a porous globule of the specific gravity 18·6. Oxide of tantalum was fused and reduced. The metal was of a yellowish colour and brittle. Oxide of cerium was fused without being reduced. This was the case also with oxide of titanium. Oxide of tungsten was reduced and fused. The metal was grey and very heavy. Oxides of molybdenum and uranium were likewise fused and reduced, and both metals were brittle.

---

*Report of the National Vaccine Establishment, for the Year* 1814; *dated* 19*th June*, 1815.

*To the Right Honourable Lord Viscount Sidmouth, Principal Secretary of State for the Home Department, &c. &c. &c.*

*National Vaccine Establishment,*
*Leicester-square,* 19*th June,* 1815.

My Lord.—The Board of the National Vaccine Establishment has the honour to report to your lordship, that a greater number of individuals has been vaccinated in the course of last year than the preceding; that several thousand more charges of vaccine lymph* have been distributed to the public, whence the destructive ravages of small pox have been diminished.

It appears from the bills of mortality of London, that the deaths occasioned by small pox have decreased in a larger proportion than one-fourth, six hundred and thirty-eight having fallen victims to that malady during the last year, eight hundred and ninety-eight during the former. Large indeed is this melancholy catalogue, which is attributable to the dissemination of variolous matter by a few interested individuals, who, from sordid motives, con-

* At the different stations 4,686 persons have been vaccinated, and 32,190 charges of lymph have been distributed.

tinue tinue the practice of inoculating with small pox virus, and diffusing this fatal disease through the metropolis.

With the view of augmenting the benefits of this establishment, the board has lately appointed a class of extraordinary vaccinators, in addition to the stationary surgeons of respectability, who have voluntarily stepped forward to contribute their assistance gratuitously, compose this class, from which it is intended hereafter to elect the stationary vaccinators.

Another class, denominated corresponding vaccinators, has also been established, from which a very material extension of the benefits to be derived from the Vaccine Institution is confidently expected. Each person will in his own neighbourhood be a point, from which the practice will continually diverge, and through whom any communication of importance may at once be made to this board.

The stationary and extraordinary vaccinators must reside in London, or the suburbs; but the corresponding may live at any distance, or in any part of the world.

The official communications from the medical colleges of Edinburgh, Glasgow, and Dublin, evincing their confidence in vaccination, and the annihilation of small pox in the settlements of the Cape of Good Hope and of Ceylon, by its introduction, as formerly reported, have been insufficient to convince some individuals of the security against the infection of small pox; but it is to be hoped, that the strong additional facts hereafter stated, will produce the fullest conviction of its benefits in their minds.

From the official documents transmitted by the Right Honourable the Secretary of State for Foreign Affairs, to this board, respecting the effects of vaccination in the Islands of Mauritius and Bourbon, it appears that the inhabitants have been secured against the visitation of one of the severest scourges incident to the human race, as the sequel shews. In the year 1728, the small pox swept off nearly one-half of the population; in 1756, about one-fourth; in 1771 and 1772, it occasioned a comparatively less, though very great, mortality; and in 1792, it destroyed one-third; and of those who survived the disease, one-third lingered out a short and miserable existence, afflicted with dropsy, marasmus, consumption, &c. It is worthy of remark, that, in three times out of the four, the disease was introduced by slave ships. Let the contrast now be drawn between the introduction of variolous infection and vaccine inoculation.

In 1802, vaccination was introduced from the British possessions in India, but its general use was prevented by the prejudices of the people, and the lymph, after a short time, could not be procured. In 1805, it was re-introduced, and the French government seeing the necessity of regulations, framed some accordingly; but vaccination was only partially adopted, for it did not exist in many parts of the island when the British took possession of it. In 1811, the small pox re-appeared in the island, and about 220 persons became infected, of whom thirty died. The alarm excited by the progress of this disease, prompted his Excellency Governor Farquhar

Farquhar to issue his mandate, compelling all the inhabitants to be immediately vaccinated, which energetic measure at once arrested the progress of small pox.

In 1813, an opportunity was offered of putting to the test the security of vaccination, by a slave (who came from the Island of Madagascar, and was afflicted with the confluent form of small pox) having been landed and received into the hospital; many slaves and other vaccinated persons, were exposed to the infection, but no one became the subject of the disease.

From the introduction of vaccination in 1802, to the 28th of February, 1814, it is computed that 200,000 persons have been vaccinated; and the medical practitioners unanimously declare, that no instance has occurred of small pox being contracted after regular vaccination.

In the Island of Bourbon the calamitous effects of variolous disease, and the beneficial consequences of vaccination, though detailed in a more abridged form, as forcibly corroborate the utility of vaccine inoculation, from its having banished the small pox from that settlement.

Some remarks are added on the difficulty of preserving the vaccine lymph, and the means taken by the board; after which follow the accounts given in our former Journal of prosecutions for exposing children under small pox inoculation, and on the necessity of a public receptacle for those who persevere in that practice. To this a note is added signifying that "the *Small Pox Hospital* has been lately purchased for the use of the sick poor afflicted with fevers." That a public establishment should be discontinued at the moment when by such respectable authority it is pronounced to be most wanted, appeared to us so strange, that we could not help enquiring into the *truth* of this statement. We find that the governors are negociating to dispose of an additional wing, the spacious establishment being now thought sufficient for all their purposes; and that of this they have no intention to alienate any part.—*Quære*. How could such a mis-statement have been foisted into a solemn document, signed by the heads of two royal medical establishments, and presented as an official paper to the secretary of state?

---

The *Courier of July* 25, 1815, contained the following:—

At the Exeter Assizes; the King *versus* Evans and Wife.—Mr. Serjeant Lens stated this to be an indictment preferred against the defendants for having inoculated and exposed in public an infant, their child, at a village in this county, while it had an infectious disease called the small pox; but, it not appearing in evidence that the child was so exposed as to endanger any of his Majesty's subjects by infection,—Mr. Justice Chambre said, this was the most absurd indictment he ever saw brought into a court of justice; for the defendants had only inoculated their infant in their own dwelling, which they had a right to do if they thought fit, in preference to vaccination; there was no pretence for an indictment for it. Those who first introduced inoculation were great benefactors to mankind. Of late years, the general opinion was much in in favour of vaccination, but, in this matter, the parents of infants had a right to judge for themselves, whether they would adopt vaccination, or pursue the old mode of inoculation. Mr. SERJEANT PELL said, this prosecution was instituted and pursued upon motives the most reprehensible.—The defendants were instantly acquitted.

---

*Linnæan Society.*—On Tuesday, the 6th of June, a paper by Dr. BENJAMIN SMITH BARTON was read, giving an account of a singular bird lately observed in the United States of America, which he considers as a new species of tantalus.

On Tuesday, the 20th of June, a paper by Mr. J. MURRAY was read, containing experiments on the application of vegetable poisons to animals. He laid bare the crural nerve and muscle of the hind leg of a frog, and, applying the poisonous juice to the part, tried whether the muscle could be excited by a galvanic battery. Opium destroyed the excitability in five minutes; but the addition of citric acid restored it again. Tincture of digitalis produced no effect. A considerable number of similar experiments were related.

At the same meeting, a paper by Mr. BICHENO was read, describing three native species of orchis, hitherto very frequently confounded together.

At the same meeting, a Latin paper by Sir JUSTLY GREEN, Bart. was read, describing forty species of phascum.

---

The Apothecaries' Bill is at length passed, and that respectable body is likely to assume the rank they so justly merit. The bill will probably undergo some further alterations at a future session, as it is confessed by the Lord Chancellor not to be entirely free from the objections mentioned by Lord Stanhope. It is altogether prospective, taking no notice of those who are already engaged in practice, but requiring every future apothecary and apothecary's assistant to undergo an examination at their hall by examiners appointed by their court.

---

We take no notice of the Surgeon's Bill, or the bill proposed by the Vaccine Board, as they were rejected by the Lords.

---

We have to lament the premature death of Dr. SATTERLEY, a gentleman whose promising abilities and engaging manners had secured him at an early period of life a very large and respectable share of practice. His complaint was an abscess of the liver, the symptoms of which were so mild as not to be suspected till at an advanced period. He had married an amiable young lady a few weeks before his death. He is likely to be succeeded in the Middlesex Hospital by Dr. SOUTHEY, brother to the poet-laureat.

---

Mr. Dick has published the following observations, which we transcribe from the Annals of Philosophy:—" A quantity of timber being felled at Fountain-hall, by Tranant, a wright, who had made some purchases, came to take his trees away, and amongst the rest a beech, which had grown with a smooth, straight, unbranched, stem of about thirty feet high, above which it divided into two large limbs. As this tree was lying on the ground, the wright and his his man set about cross cutting it with a saw just below the cleft, when, to their surprise, the stem was no sooner divided than a large toad crept out of a circular hole, the upper and smaller part of which had been cut off by the saw. As far as I can make out from conversation with the man above alluded to, the tree had all the appearance of being quite solid above; yet I have no doubt that some slight, though perhaps almost imperceptible, communication, must have existed from the fork into the hole where the toad was lodged; and I am the more satisfied of this from the account which the man gives of the appearance of the interior of the hole, which seemed to be sheathed all round with something resembling bark.

"But the curious query arising from this fact is, how came a toad to be lodged so high? The toad has no power of crawling perpendicularly so as to have ascended the smooth bark of a straight tree to such a height. I know from my own observations that trees grow in altitude in two ways: 1st. Something is annually added to the height of the tree by the new shoots: and, 2dly, in addition to this mode of increment, the whole tree seems to stretch itself yearly out of the ground, throughout its entire length, to a very considerable extent, as I have proved by measuring the height of knots upon trees at different periods. But with all this I do not think it very rational to suppose that the cleft in question, which may have once extended quite down to the hole, could have ever existed so near the ground of a size sufficient to have admitted of a toad crawling into it. The only way in which it appears to me that this circumstance can be accounted for, is by supposing that the spawn, after being removed from the female by the obstetrical aid of the male toad, must have been transported and dropt into the cleft of the tree by some bird.

"As to what Pennant and others say of the obstetrical aid afforded by the male to the female toad, I am lead to suspect that the object of the operation is more for the purpose of impregnating the spawn as it is dragged from the female than any thing else. It appears to be the same with frogs. In the course of a solitary walk in the beginning of last March, my attention was excited by an uncommon commotion in a shallow pool of water not much more than four feet square. My approach to ascertain the cause being rather too hasty, I had only time to observe it was occasioned by a parcel of frogs, when immediately on my advance they disappeared under water, concealing themselves beneath a quantity of spawn already floating on the surface. Having placed myself behind an adjoining hedge, through which I could perfectly see the pool, being about two yards from its surface, and at the same time without giving any disturbance to its inhabitants, I remained quiet for more than a quarter of an hour. At length, when my patience was nearly exhausted, I saw the head of a frog rise above water; and on a closer inspection I perceived underneath the head of another one, which seemed to be embraced by the first. In this way they silently raised themselves pair by pair, till there were not less than fifty or sixty pairs of them in the small space I have already mentioned. In a short short time the little pool was all in action. Those frogs which were mounted on the backs of the others seemed to be busily employed with their hinder feet and legs, whilst the fore legs of each firmly embraced the body of his mate. In some too a mass of spawn seemed to move after the pair as they altered their position in the pool. These violent exertions of what I took to be the males continued without any intermission, and with so much force as very considerably to agitate the little pool for some time, until the noise of a person passing on horseback alarmed them, and they were all again under water in a moment. From the hinder parts of what I took to be the female frogs having been so much under water, I could not positively assert the fact, but I had not a doubt, from the nature of the motions I saw, that the animals were engaged in an operation similar to that which is ascribed to the toad; and I was confirmed in this belief by observing on my return, five or six hours afterwards, that the quantity of spawn had been nearly doubled; and, though I approached the pool with the utmost caution, I could not see a single frog, and had every reason to think, from a careful examination of the shallow pool, that they were all gone."

On the subject of the *entombed* toad, we cannot help remarking that it is by no means ascertained that all animals in a torpid state respire, or require air. There is even reason to believe, that the hog buried under the cliff at Dover for several months during the winter was excluded from air.

---

It appears from a note in Dr. Holland's Travels, p. 411, that at Athens the thermometer sometimes rises in summer to 104°, and that in winter it falls as low as 28°. In July the thermometer is frequently above 90°. The average quantity of rain that falls is stated at only 21 or 22 inches; probably French, as the observer, M. Fauvel, was a Frenchman.

---

*Pemphigus* is a disease of somewhat rare occurrence, and we believe has not very frequently been observed to prevail epidemically. Mons. Petiet has seen it assume this character in the village of Batterans, containing about 294 inhabitants, without any apparent cause for such an anomaly. It came on with the usual symptoms of fever, in addition to which the patients were affected with general tumefaction, and an insupportable degree of itching. On the third day, vesicles, varying in size from that of a grain of hemp-seed to that of a walnut, appeared on every part of the surface of the body, but more particularly on the abdomen, the arms, and the thighs. They contained a transparent and inodorous fluid, were easily broken, and left a violet brown spot upon the skin, which soon disappeared. In slight cases the fever sensibly abated after the appearance of the eruption, and the cure was effected in a week. In some cases, typhoid symptoms supervened, but the author does not state that the disease terminated fatally in any instance. He gave an emetic on the accession of the disease; and, with respect to the after treatment, he says nothing more than that he employed the aqua ammoniæ acetatis, blisters, diffusible tonics, and camphor, in cases requiring them; but that the

 emetic emetic and nitrous draughts were generally all that was necessary. Out of 294 inhabitants, 35, principally children, were attacked with the disease.—*New England Journal.*

---

M. Zetherman has given an account of a dumbness occasioned by a calculous concretion, situated on the left of the frænum linguæ, and probably in the salivary duct. The patient was enabled to speak, (at the age of fourteen years,) after having spit out this stone, which was the size of a turkey-bean. The same gentleman found a tuft of hair in a vesicular tumour which he extirpated from the eye-lid.

---

A very curious phenomenon was lately seen at the Small Pox Hospital—a completely pie-bald child. The parents are white, and there is no tradition of any mixture of colour in the family.

---

Dr. Spurzheim has published a second edition of his Physiognomical System. The principal addition consists of remarks on those papers of Sir Everard Home given in our last Number from the Philosophical Transactions: these shall be transcribed in our next Number.

---

Dr. Young is printing a work entitled, A Practical and Historical Treatise on Consumptive Diseases; exhibiting a concise Account of the State of Medical Science in all ages.

---

*Theatre of Anatomy, Great Windmill Street.*—The winter Course of Lectures on Anatomy, Physiology, Pathology, and Surgery, by James Wilson, F.R.S. and Charles Bell, F.R.S.E. will commence on Monday, October the 2d, at two o'clock. The Demonstrations in the Dissecting Rooms will be given by Mr. Shaw, who will attend during the forenoon.

---

The following Lectures, which were formerly delivered at the *Theatre of Anatomy*, will, in future, be delivered in the *Medical Theatre, No.* 42, *Great Windmill Street:*—

1. On the Laws of the Animal Œconomy, and the Theory and Practice of Physic; by George Pearson, M.D. F.R.S. Senior Physician to St. George's Hospital.*
2. On Materia Medica, Therapeutics, and Medical Jurisprudence; by Richard Harrison, M.D. Fellow of the Royal College of Physicians, and Physician to the Northern Dispensary.
3. On Chemistry; by ——— Granville, M.D.
4. On the Theory and Practice of Surgery; by B. C. Brodie, F.R.S. Assistant-Surgeon to St. George's Hospital.

Sir Everard Home's gratuitous Lectures to the pupils of St. George's Hospital, will also be given in this Theatre.

---

Dr. Squire commenced, on the 4th of July, a Course of Lectures on the Theory and Practice of Midwifery, and the Diseases of Women and Children.

---

* Some of Dr. Pearsons's Lectures are delivered at his Theatre in George-street, Hanover-square.

### LONDON PRICES OF DRUGS.—July 21, 1815.

| | £. | s. | d. | £. | s. | d. per |
|---|---|---|---|---|---|---|
| Aloes Barbadoes, from | 22 | 0 | 0 to | 0 | 0 | 0 C. |
| ——Cape | 6 | 10 | 0.. | 7 | 0 | 0 — |
| ——Succotrina | 23 | 0 | 0.. | 0 | 0 | 0 — |
| ——Epatica or E.I. | 14 | 0 | 0.. | 16 | 0 | 0 — |
| Angelica Root | 8 | 0 | 0.. | 8 | 10 | 0 — |
| Alkanet Root | 8 | 10 | 0.. | 0 | 0 | 0 — |
| Antimony Crude | 2 | 18 | 0.. | 3 | 5 | 0 — |
| Aqua Fortis | S. | 0 | 8.. | D. | 1 | 1 lb. |
| Arrow Root, fine | 0 | 2 | 0.. | 0 | 2 | 9 — |
| ——ordinary | 0 | 0 | 9.. | 0 | 1 | 3 — |
| Arsenic, Red | 4 | 4 | 0.. | 4 | 10 | 0 C. |
| ——White | 2 | 16 | 0.. | 0 | 0 | 0 — |
| Balsam Capivi | 0 | 4 | 10.. | 0 | 5 | 0 lb. |
| ——Peru | 1 | 0 | 0.. | 1 | 1 | 0 — |
| ——Tolu | 0 | 13 | 6.. | 0 | 14 | 0 — |
| Bark Jesuits', Com. | 0 | 3 | 6.. | 0 | 4 | 0 — |
| ——Second | 0 | 6 | 0.. | 0 | 7 | 0 — |
| ——Quil or best | 0 | 8 | 0.. | 0 | 10 | 0 — |
| ——Red | 0 | 8 | 8.. | 0 | 9 | 0 — |
| ——Yellow | 0 | 2 | 0.. | 0 | 2 | 2 — |
| Borax, refined E.I. | none. | | | | | |
| ——English | 0 | 2 | 8.. | 0 | 3 | 0 lb. |
| ——unrefined or Tinc. | 6 | 10 | 0.. | 7 | 15 | 0 C. |
| Camphire, refined | 0 | 6 | 0.. | 0 | 6 | 3 lb. |
| ——unrefined | 16 | 0 | 0.. | 17 | 10 | 0 C. |
| Cantharides | 0 | 9 | 6.. | 10 | 0 | 0 lb. |
| Cardamoms (best) | 0 | 6 | 6.. | 0 | 7 | 6 — |
| Cassia Buds | 32 | 0 | 0 } | 0 | 0 | 0 C. |
| ——Fistula, W.I. | 5 | 5 | 0 } | in bond | | — |
| ——Lignea | 42 | 0 | to } | 45 | 0 | 0 — |
| Castoreum American | 2 | 10 | 0.. | 3 | 0 | 0 lb. |
| ——Russia | 11 | 11 | 0.. | 0 | 0 | 0 — |
| Castor Oil, per bottle 1½ lb. | 0 | 3 | 9.. | 0 | 4 | 0 bo |
| Cocculus Indicus | 6 | 0 | 0.. | 7 | 0 | 0 C. |
| Colocynth Turkey | none. | | | | | |
| Columbo Root | 4 | 15 | 0.. | 6 | 0 | 0 C. |
| Cream of Tartar | 5 | 10 | 0.. | 6 | 0 | 0 — |
| Gallangal East-India | uncertain | | .. | 0 | 0 | 0 C. |
| Gentian Root | 3 | 10 | 0.. | 3 | 15 | 0 — |
| Ginseng | none | | .. | 0 | 0 | 0 lb. |
| Grains of Guinea | 6 | 10 | 0.. | 0 | 0 | 0 C. |
| Gum Ammo. Drop | 50 | 0 | 0.. | 0 | 0 | 0 — |
| ——Lump | 5 | 0 | 0.. | 7 | 0 | 0 — |
| ——Animi | 3 | 10 | 0.. | 7 | 10 | 0 — |
| ——Arabic, E.I. | 2 | 0 | 0.. | 4 | 0 | 0 — |
| ——Turkey, Fine | 9 | 0 | 0.. | 9 | 10 | 0 — |
| ——Barbary | 5 | 5 | 0.. | 0 | 0 | 0 — |
| ——Assafœtida | 10 | 0 | 0.. | 15 | 0 | 0 — |
| ——Benjamin | 15 | 0 | 0.. | 60 | 0 | 0 — |
| ——Gambogium | 28 | 0 | 0.. | 30 | 0 | 0 — |
| ——Copal, scraped | 0 | 5 | 9.. | 0 | 6 | 0 lb. |
| ——Galbanum | 28 | 0 | 0.. | 0 | 0 | 0 C. |

| | £. | s. | d. | £. | s. | d. per |
|---|---|---|---|---|---|---|
| Gum Guaiacum, from | 0 | 2 | 6 to | 0 | 3 | 0 lb. |
| ——Mastic | 0 | 4 | 6.. | 0 | 4 | 9 — |
| ——Myrrh | 15 | 0 | 0.. | 20 | 0 | 0 C. |
| ——Olibanum | 8 | 0 | 0.. | 11 | 0 | 0 — |
| ——Oppoponax | 80 | 0 | 0.. | 0 | 0 | 0 — |
| ——Sandrac | 8 | 0 | 0.. | 8 | 5 | 0 — |
| ——Senegal | 6 | 15 | 0.. | 7 | 0 | 0 — |
| ——Tragacanth | 26 | 15 | 0.. | 27 | 0 | 0 — |
| Jalap | 0 | 5 | 6.. | 0 | 5 | 9 lb. |
| Ipecacuanha | 0 | 14 | 6.. | 0 | 15 | 0 — |
| Isinglass Book | 0 | 9 | 0.. | 0 | 10 | 0 — |
| ——Leaf | 0 | 7 | 10.. | 0 | 8 | 6 — |
| ——Long Staple | 0 | 9 | 0.. | 0 | 10 | 6 — |
| ——Short Staple | 0 | 8 | 0.. | 0 | 8 | 6 — |
| Manna Flakey | 0 | 5 | 10.. | 0 | 6 | 6 — |
| ——Sicily in Sorts | 0 | 0 | 0.. | 0 | 0 | 0 — |
| Musk China | 0 | 14 | 0.. | 0 | 16 | 0 oz. |
| Nux Vomica | 2 | 0 | 0.. | 2 | 2 | 0 C. |
| Oil of Vitriol | 0 | 0 | 3¾ | 0 | 0 | 4 lb. |
| Opium, East-India | none | | .. | 0 | 0 | 0 — |
| ——Turkey | 1 | 4 | 6.. | 0 | 0 | 0 — |
| Pink Root | 0 | 4 | 0.. | 0 | 4 | 6 — |
| Quicksilver | 0 | 5 | 10.. | 0 | 0 | 0 — |
| Rhubarb, East-India | 0 | 9 | 0.. | 0 | 13 | 0 — |
| ——Russia | 0 | 18 | 0.. | 1 | 0 | 0 — |
| Saffron, Spanish | 2 | 16 | 0.. | 3 | 0 | 0 — |
| ——French | 1 | 18 | 0.. | 2 | 0 | 0 — |
| Sago | 6 | 15 | 0.. | 8 | 10 | 0 C. |
| Sal. Ammoniac | 10 | 10 | 0.. | 11 | 0 | 0 — |
| Sarsaparilla | 0 | 2 | 3.. | 0 | 4 | 9 lb. |
| Sassafras | 3 | 0 | 0.. | 3 | 10 | 0 T. |
| Scammony, Aleppo | 1 | 14 | 0.. | 1 | 15 | 0 lb. |
| ——Smyrna | 0 | 18 | 0.. | 0 | 0 | 0 — |
| Senna, Alexandria | 0 | 4 | 0.. | 0 | 4 | 3 — |
| Seeds, Anni Alicant | 4 | 15 | 0.. | 5 | 0 | 0 C. |
| ——Coriander English | 0 | 14 | 0.. | 0 | 0 | 0 — |
| ——Cummin | 5 | 1 | 0.. | 0 | 0 | 0 — |
| ——Fenugreek | 1 | 15 | 0.. | 0 | 0 | 0 — |
| Shellack | 7 | 10 | 0.. | 10 | 15 | 0 — |
| Sticklack | 3 | 10 | 0.. | 7 | 0 | 0 — |
| Snake Root | 0 | 15 | 6.. | 0 | 0 | 0 lb. |
| Soap, Castile or Spanish | 9 | 0 | 0.. | 10 | 0 | 0 C. |
| Spermaceti, refined | 0 | 2 | 2.. | 0 | 0 | 0 lb. |
| Tamarinds, West-India | 9 | 0 | 0.. | 10 | 0 | 0 C. |
| Tapioca, Lisbon | 0 | 0 | 9.. | 0 | 1 | 0 lb. |
| Turmeric, Bengal | 4 | 10 | 0.. | 4 | 15 | 0 C. |
| ——China | 6 | 10 | 0.. | 7 | 0 | 0 — |
| ——West-India | 3 | 10 | 0.. | 4 | 4 | 0 — |
| Verdigris, Wet | 0 | 3 | 6.. | 0 | 0 | 0 lb. |
| ——Dry | 0 | 6 | 6.. | 0 | 7 | 0 — |
| ——Crystallized | 0 | 9 | 0.. | 0 | 9 | 6 — |
| Vitriol, Roman | 0 | 0 | 7.. | 0 | 0 | 0 — |
| ——Foreign white | 2 | 10 | 0.. | 0 | 0 | 0 C. |

*Price of Vials per Gross.*—8 oz. 70s.—6 oz. 58s.—4 oz. 47s.—3 oz. 43s.—2 oz. 36s.—1 oz. 30s. — ½oz. 24s.

Lisbon Leeches, 30s. per hundred.—True French ditto, 55s.
Essential Salt of Lemons, 4s. 6d. per dozen.

### METEOROLOGICAL REGISTER.

***From June the 25th, to July the 25th,* 1815.**

**Kept by C. BLUNT, Philosophical Instrument Maker, No. 38, Tavistock-Street, Covent-Garden.**

| Moon. | Day. | Wind. | Barometrical Pressure. Max. | Min. | Mean. | Temperature. Max. | Min. | Mean. | |
|---|---|---|---|---|---|---|---|---|---|
| | 26 | W | 30·06 | 30·03 | 30·045 | 69 | 50 | 59·5 | Fair |
| | 27 | SW | 30·10 | 30·07 | 30·077 | 71 | 52 | 61·5 | Fair |
| | 28 | SW | 30·20 | 30·16 | 30·18 | 73 | 51 | 62 | Fair |
| ◑ | 29 | W | 30·25 | 30·18 | 30·2 | 72 | 51 | 61·5 | Fair |
| | 30 | NW | 30·17 | 30·08 | 30·115 | 70 | 50 | 60 | Fair |
| | 1 | NNW | 30·05 | 30·05 | 30·05 | 69 | 51 | 60 | Fair |
| | 2 | W | 30 | 30 | 30 | 71 | 51 | 61 | Fair |
| | 3 | W | 30 | 29·95 | 29·952 | 71 | 52 | 61·5 | Fair |
| | 4 | NW | 30 | 29·98 | 29·985 | 71 | 50 | 60·5 | Fair |
| | 5 | NW | 30·07 | 30·05 | 30·055 | 70 | 49 | 59·5 | Fair |
| ● | 6 | W | 30 | 29·93 | 29·962 | 71 | 50 | 60·5 | Fair |
| | 7 | W | 29·98 | 29·93 | 29·963 | 72 | 49 | 60·5 | Fair |
| | 8 | NW | 30·09 | 30·05 | 30·093 | 72 | 49 | 60·5 | Fair |
| | 9 | NW | 30·05 | 30·05 | 30·05 | 74 | 46 | 60 | Fair |
| | 10 | N | 30·14 | 30·13 | 30·135 | 80 | 51 | 65·5 | Fair |
| | 11 | N | 30·13 | 29·97 | 30·045 | 82 | 51 | 66·5 | Fair |
| | 12 | S | 30·05 | 30·03 | 30·045 | 90 | 52 | 71 | Fair |
| ◐ | 13 | S&W p.m. | 30·03 | 30 | 30·007 | 85 | 56 | 70·5 | Fair |
| | 14 | SW | 30·06 | 30·04 | 30·05 | 83 | 58 | 70·5 | Fair, Rain during Night |
| | 15 | W | 30·02 | 29·9 | 29·952 | 82 | 59 | 70·5 | Rain |
| | 16 | NW | 29·88 | 29·88 | 29·88 | 77 | 56 | 66·5 | Fair |
| | 17 | W | 29·86 | 29·80 | 29·83 | 80 | 53 | 66·5 | Fair |
| | 18 | NW | 29·80 | 29·80 | 29·80 | 74 | 52 | 63 | Fair |
| | 19 | W | 29·61 | 29·56 | 29·587 | 67 | 50 | 58·5 | Fair |
| | 20 | N | 29·86 | 29·75 | 29·802 | 65 | 48 | 56·5 | Much rain in heavy showers |
| ○ | 21 | NW | 29·92 | 29·90 | 29·91 | 68 | 48 | 58 | ditto ditto |
| | 22 | NW | 29·90 | 29·88 | 29·885 | 70 | 51 | 60·5 | Fair |
| | 23 | W | 29·92 | 29·92 | 29·92 | 74 | 51 | 62·5 | Showers, with fair intervals |
| | 24 | N | 30·02 | 29·98 | 30 | 77 | 56 | 66·5 | Fair, Rain at Night |
| | 25 | NW | 30·09 | 30·06 | 30·08 | 72 | 57 | 65 | Fair |

**RESULTS.**

Mean barometrical pressure of the month 29·991
Max. June 29th, 30·25, wind at W
Min. July 19th, 29·56, ——— W

Mean temperature of the month 62·883 deg.
Max. July 12th, 90, wind at S
Min. —— 9th, 46, ——— NW

*Scale exhibiting the prevailing Winds during the Month.*

| N | NE | E | SE | S | SW | W | NW |
|---|---|---|---|---|---|---|---|
| 4 | 0 | 0 | 0 | 2 | 3 | 10 | 11 |

| | Mean barometrical pressure. | Mean temperature. |
|---|---|---|
| From the last quarter on the 29th June, to the new moon on the 6th of July | 30·051 | 60·57 |
| ——— new moon on the 6th, to the first quarter on the 13th | 30·184 | 63·5 |
| ——— first quarter on the 13th, to the full moon on the 21st | 29·863 | 65·062 |

REPORT

### REPORT OF DISEASES.

The diseases which came under the Reporter's care during the last month were rather numerous, considering the season of the year, and the fineness of the weather. Some slight cases of fever have occurred, and one of those was cured by cold affusion. In a case of rubeola, pneumonia supervened and required bleeding. Several cases of pertussis were alleviated by emetics and nauseating medicines. Dyspepsia is generally a frequent complaint during the warm season; in ten cases it was cured by cascarilla, steel, and cathartics; in five instances, where pyrosis was the leading symptom with constipated bowels, liquor potassæ and calcined magnesia were useful. Gastrodynea, except in one instance, was uniformly relieved by the oxyd of bismuth. Several cases of idiopathic cephalalgia, one of them of seven years, and most of the others of one year's standing, yielded to the application of electricity without the aid of any medicine. This powerful agent also proved useful in some instances of intestinal and glandular obstruction. In a case of hydrothorax, digitalis and small doses of murias hydrargyri, produced a salutary change. A severe case of dyspnœa, which had been treated for six years as confirmed asthma, is mending under a mercurial course and nitric acid. Out of five patients affected with scarlatina, two had the complaint very severely. Two children, affected with hooping cough, were attacked with confluent small pox, of which disease a third child lay dead in the same chamber. In an instance of paralysis of the arm, purging produced very sensible relief. Six cases of menorrhagia were cured by using an injection of twenty grains of ipecacuanha, twenty drops of laudanum, and a hundred of tincture of black henbane in a pint of gruel; the internal remedies were one or two emetics, nauseating doses of ipecacuanha; and, lastly, sulphuric acid. It is singular, that, in six instances of confirmed phthisis, all the patients were tailors. None of them will recover. Some cases of incipient phthisis are improving by means of small bleedings, digitalis, blisters, and purgatives.

Besides the chronics already enumerated, several others have occurred, which did not offer any thing particularly worthy of remark. Amongst these were various affections (chiefly diarrhœa) of the bowels, hepatic disease, rheumatism, and catarrh. An unfortunate deserted woman, destitute of means, died a few days after parturition; she had phlegmasia dolens with considerable fever, delirium, and diarrhœa. Her infant also perished. Depression of mind was a marked and distressing symptom in the mother from the first.

MONTHLY

